# Correction: A dual-clock-driven model of lymphatic muscle cell pacemaking to emulate knock-out of Ano1 or IP_3_R

**DOI:** 10.1085/jgp.20231335506112024c

**Published:** 2024-07-01

**Authors:** Edward J. Hancock, Scott D. Zawieja, Charlie Macaskill, Michael J. Davis, Christopher D. Bertram

Vol. 155, No. 12 | https://doi.org/10.1085/jgp.202313355 | October 18, 2023

The authors regret that, in the original article, the green curves in the right panels of Figs. S1 and S2 and Videos 1 and 2 appeared slightly higher than they should have. The corrected figures appear here, and the figure and video legends remain unchanged. The conclusions of the paper are not affected by these errors, and all discussion of the data presented is still correct. The errors appear in print and in PDFs downloaded before June 18, 2024.

**Figure S1.**
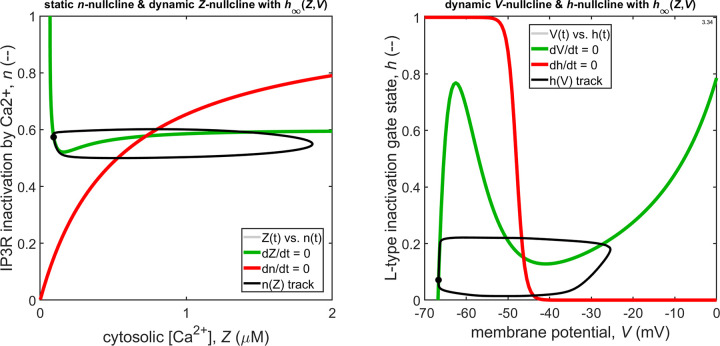
The animation, intended for running on endless loop, shows how four variables *Z*, *n*, *V* and *h* of the model vary over the course of a control pace-making cycle.

**Figure S2.**
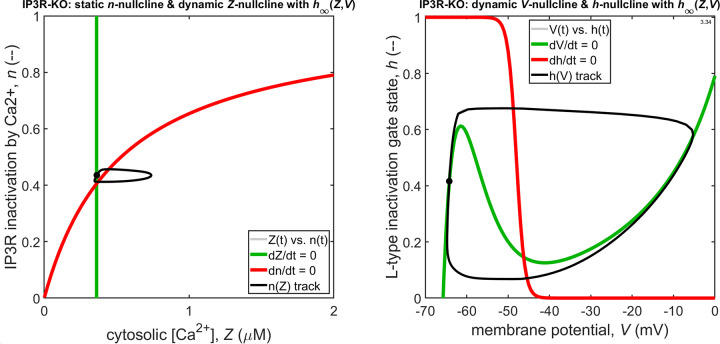
As above, but now for the model with knock-out of store IP_3_ receptors.

